# Metabolomics study of the effects of zinc sulfate in minimal hepatic encephalopathy

**DOI:** 10.1038/s41598-026-43902-0

**Published:** 2026-03-17

**Authors:** Tao Zhang, Qi Chen

**Affiliations:** 1 Department of Radiology, Jinshan Hospital, Fudan University, Shanghai, 201508 China; 2 Department of Medical Ultrasound, Jinshan Hospital, Fudan University, Shanghai, 201508 China

**Keywords:** Minimal hepatic encephalopathy, Zinc sulfate, Metabolomics, Nuclear magnetic resonance, Cognitive impairment, Biochemistry, Diseases, Neuroscience

## Abstract

Minimal hepatic encephalopathy (MHE) is a cognitive, motor, and sleep-related disorder resulting from liver impairment, often linked to elevated ammonia levels. Zinc deficiency is common in patients with cirrhosis and has been associated with cognitive dysfunction. Zinc supplementation has shown promise in improving MHE, but the underlying metabolic mechanisms remain unclear. To investigate the therapeutic effects of zinc sulfate on metabolic changes in the striatum of MHE rats using ^1^H-NMR-based metabolomics. We established a rat model of MHE using partial portal vein ligation and administered zinc sulfate to a subset of rats. A control group was included for comparison. Behavioral assessments of spatial learning and memory were performed using the Morris water maze (MWM). Striatum metabolites were analyzed through ^1^H-NMR spectroscopy, and key metabolic pathways were identified using statistical analyzes. Zinc supplementation improved cognitive performance in the MHE rats, evidenced by reduced escape latency in the MWM. Metabolomics analysis identified 47 metabolites, with 10 key metabolites showing significant differences between MHE and control groups. Zinc supplementation normalized several disrupted pathways, including those related to glycolysis, glutamine metabolism, and BCAA metabolism. Key metabolites affected by zinc included lactate, alanine, glutamate, and branched-chain amino acids. Zinc sulfate supplementation alleviates cognitive impairments in MHE rats by restoring disrupted metabolic pathways, including nitrogen metabolism. The findings suggest that zinc plays a therapeutic role in improving brain function in MHE.

## Introduction

Hepatic encephalopathy (HE) is a neurological syndrome resulting from metabolic dysfunction due to liver impairment or porto-systemic shunting^1^. Minimal hepatic encephalopathy (MHE) represents a sub-type of HE characterized by cognitive, motor, and sleep-related abnormalities without overt clinical symptoms^2^. The pathogenesis of MHE is multifaceted and not fully understood, with elevated blood ammonia levels recognized as a primary contributor^1^. Consequently, therapies targeting ammonia reduction are pivotal for managing HE^2^.

Numerous studies have identified trace element deficiencies, notably zinc, as common in patients with cirrhosis^3,4^. Zinc serves as a crucial co-enzyme for various metabolic enzymes and plays a fundamental role in biochemical processes^5^. Deficiencies in zinc have been associated with metabolic, behavioral, and cognitive disorders^6^. Oral zinc supplementation has shown promise in improving MHE^7^, yet the metabolic mechanisms underlying its efficacy remain under-explored.

Nuclear magnetic resonance spectroscopy (NMR) enables continuous monitoring of metabolites such as amino acids and fatty acids in bodily fluids and tissues^8^. Metabolomics, facilitated by advancements in analytical techniques, has emerged as a valuable approach in medical research for identifying disease biomarkers through comprehensive metabolic profiling^9^.

In this study, we established a rat model of MHE and employed ^1^H-NMR-based metabolomics to investigate metabolic changes in the rat striatum with and without zinc supplementation. By comparing metabolite profiles, our aim is to elucidate the therapeutic mechanisms of zinc in MHE metabolism.

## Methods

### Research ethics

This study was approved by the Institutional Review Board of the Department of Institional Animal Care and USE Committee o SHZY(Permit No. SHZY-20230614E). All procedures were in accordance with the guidelines of the Institutional Animal Care and Use Committee of SHZY (IACUC) Guide for Care and Use of Laboratory Animal.

### Laboratory animals and models

A total of 72 male Sprague-Dawley rats (8 weeks old, weighing 250–280 g at baseline) were allocated into three groups: MHE rats (*n* = 24), ZN rats (MHE treated with zinc sulfate, *n* = 24), and CN rats (sham operation controls, *n* = 24). Body weights shown represent measurements taken at the time of euthanasia (Table 1, day 61). Surgical procedures were performed under aseptic conditions. Rats were anesthetized with isoflurane (5% for induction, 2–3% for maintenance) delivered via inhalation. Anesthetic depth was monitored throughout the procedure by assessing respiratory rate and pedal withdrawal reflex. Body temperature was maintained at 37 °C using a heating pad during surgery. After completion of the MWM test, rats were deeply anesthetized with 5% isoflurane in oxygen for induction, followed by maintenance at 2–3% isoflurane via inhalation using a nose cone/anesthesia chamber. MHE in rats was induced by partial ligation of the portal vein following procedures outlined in a previous study^10^. Zinc sulfate (30 mg/kg/day elemental zinc) was administered to ZN rats after partial portal vein ligation, prepared by dissolving 150 mg of zinc sulfate in 500 mL of deionized water^7^. Zinc sulfate doses were adjusted weekly based on the rats’ body weight and water intake. CN rats received deionized water without any additional treatment.

**Table 1:** The body weight, water consumption, feed consumption, and escape latency in the Morris water maze.

	CN (*n* = 24)	ZN (*n* = 24)	MHE (*n* = 24)	*P*-value*	*P*-value#
Test1 (s)	55.09 ± 5.71	59.50 ± 1.99	59.94 ± 0.27	< 0.001	0.294
Test2 (s)	41.23 ± 9.45	53.05 ± 9.10	55.75 ± 6.30	< 0.001	0.24
Test 3 (s)	32.84 ± 8.25	40.28 ± 6.10	48.42 ± 6.96	< 0.001	< 0.001
Test4 (s)	17.89 ± 8.88	24.62 ± 10.06	42.91 ± 10.93	< 0.05	< 0.001
Weight (g)	371.2 ± 12.8	370.1 ± 18.0	371.7 ± 16.5	0.819	0.759
Water (mL)	36.45 ± 3.22	31.17 ± 2.41	34.54 ± 3.06	< 0.001	< 0.001
Feed (g)	33.17 ± 3.34	29.43 ± 2.41	30.74 ± 2.60	< 0.001	0.077

*MHE vs. CN, # MHE vs. ZN.

Rats were housed under standard laboratory conditions with a 12-hour light/dark cycle and ad libitum access to food and water. Surgical procedures were performed under aseptic conditions, and zinc sulfate supplementation was administered orally for 56 days.

### Behavioral assessments

Behavioral testing in the Morris water maze (MWM) commenced 57–61 days after supplementation initiation to evaluate spatial learning and memory in the experimental groups. The MWM consists of a circular pool filled with opaque water where a hidden platform is located. Each rat underwent 4 trials per day for 4 consecutive days. Spatial learning and memory were assessed by recording the time taken to locate the platform (latency). The average latency of the 4 trials per day was used as the final latency.

### Sample preparation, blood ammonia measurements

After completion of the MWM test, euthanasia was performed on the rats using intravenous injection of KCl (1–2 mg/kg) through the tail vein under deep anesthesia induced by isoflurane. Brain tissues were extracted, rinsed with cold saline, and dried with filter papers. The striatum was dissected on a frozen plate following the rat brain’s anatomical map. Samples were promptly preserved in liquid nitrogen and subsequently transferred to a -80 °C freezer for subsequent analysis.

Blood samples were collected from the tail vein before euthanasia and centrifuged at 3000×g for 10 min to obtain serum. Serum ammonia levels were determined using commercial assay kits (Nanjing Jiancheng Bioengineering Institute, Nanjing, China) according to the manufacturer’s protocols.

### ^1^H-NMR metabolomics analysis

For ^1^H-NMR metabolomics analysis, samples weighing 10–20 mg were mixed with 0.6 mL ice-cooled extraction solution (CH_3_OH = 2:1). After vortexing for 1 minute, the sediments were homogenized using a tissue crusher (30 Hz, 90 s). The samples were then centrifuged (4 °C, 10,000×g, 10 min). The supernatant was taken, evaporated and freeze-dried for 24 h. The resulting solid residues were dissolved in 600 µL of buffer solution (0.1 M NaH_2_PO_4_/K2HPO_4_, pH 7.4). D2O (99.96% deuterium-enriched containing 0.05% trimethylsilylpropionic acid) served as a chemical shift reference. Subsequently, the samples were transferred into 5 mm NMR tubes.

^1^H-NMR spectroscopy was conducted using a 600 MHz NMR spectrometer (AVANCE III, Bruker, Germany) employing the Carr-Purcell-Meiboom-Gill (CPMG) pulse sequence with water suppression. The key parameters included: pulse angle = 90°; water presaturation during the relaxation delay = 2.5 s; pulse width = 10 ms; number of sampling points = 32 K; spectral width = 20 ppm; sampling time = 1.36 s; number of scans = 64; and temperature = 298 K.

### ^1^H-NMR data processing

The ^1^H-NMR free induction decays data were processed following a previously reported pipeline^11,12^. This involved a sequence of steps: group delay correction, solvent suppression, apodization, Fourier transform, zero-order phase correction, internal referencing, baseline correction, zeroing negative values, warping, window selection, bucketing, water region removal, zone aggregation, and normalization. Metabolite identification and quantification relied on a spectroscopy library of 191 pure metabolites, excluding those within 2 standard deviations of background noise from further analysis.

Supervised analysis utilized sparse projection to latent structures discriminant analysis (sPLS-DA) with 5-fold cross-validation for pattern recognition of significantly affected metabolites in MHE vs. ZN rats and MHE vs. CN rats. Key metabolites were defined by a variable influence on projection (VIP) score > 1 and *P* < 0.05 in each comparison.

### Enrichment analysis

Enrichment analysis of these key metabolites was performed. The top-ranked significant biological pathways were subsequently visualized based on the KEGG database (https://www.kegg.jp/).

### Statistical analysis

Statistical analyzes were conducted using R (Version 4.2.2; http://www.r-project.org/). Normality and variance homogeneity of the data were assessed with ANOVA, followed by false discovery rate (FDR) correction if assumptions were met. In cases where normality or variance homogeneity assumptions were not met, the Mann-Whitney U test was employed, also followed by FDR correction. For the analysis of metabolite data, the “mixOmics” package was utilized for sPLS-DA and VIP score calculations. Enrichment and pathway analyzes were performed using the “FELLA” package. A significance level of *P* < 0.05 was considered statistically significant.

## Results

### General situation

**Fig. 1:**
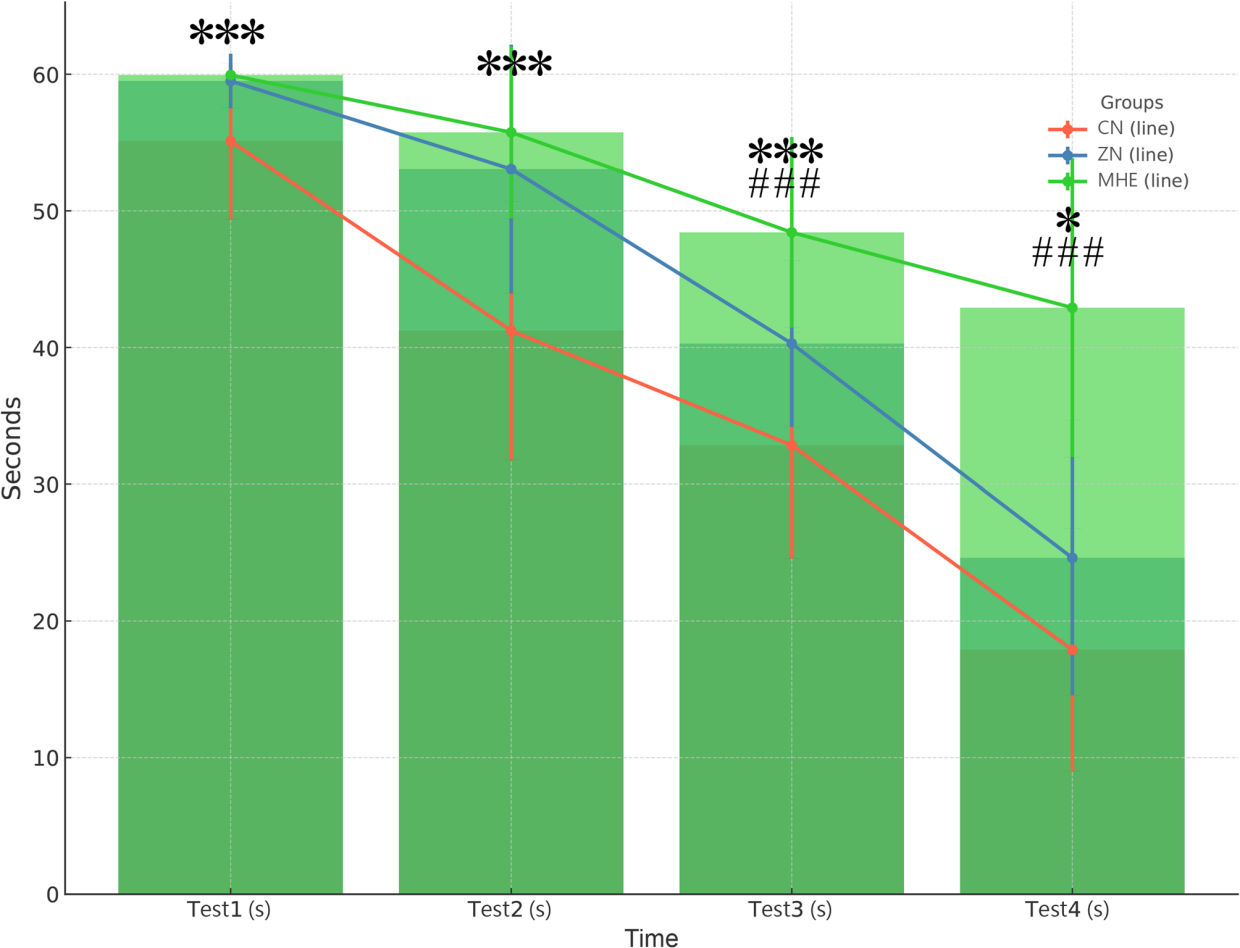
Escape latency in the morris water maze (MWM) test across four time points for control (CN), minimal hepatic encephalopathy (MHE), and zinc-supplemented (ZN) rat groups. The graph shows mean escape latency in seconds with error bars indicating standard deviation. Significant prolongation of escape latency was observed in the MHE rats compared to controls, indicating impaired spatial learning and memory. Zinc supplementation in the ZN group resulted in shorter latencies compared to untreated MHE rats, suggesting improved cognitive performance. The MHE group consistently showed the longest escape latencies across all four test sessions, while the ZN group demonstrated intermediate performance between the CN and MHE groups, highlighting the potential cognitive benefits of zinc supplementation in MHE. *P < 0.05, **P < 0.01, **P < 0.001 vs. CN; #*P* < 0.05, ##*P* < 0.01, ###*P* < 0.001 vs. MHE.

Table 1 presents the body weight, water consumption, feed consumption, and escape latency in the Morris water maze (MWM) for the three groups. There were no significant differences in body weight. A reduction in food and water consumption was observed in the ZN and MHE rats compared to the CN rats. Significant prolongation of escape latency in the MWM was observed in the MHE rats, with a shorter latency observed after zinc supplementation (Fig. 1).

Compared with the CN group (45.3 ± 5.2), MHE rats (88.7 ± 10.1) exhibited significantly elevated ammonia (*P* < 0.001) levels. Zinc supplementation significantly reduced serum ammonia levels (56.4 ± 7.3, *P* < 0.01).

### Metabolite identification and quantification

**Fig. 2:**
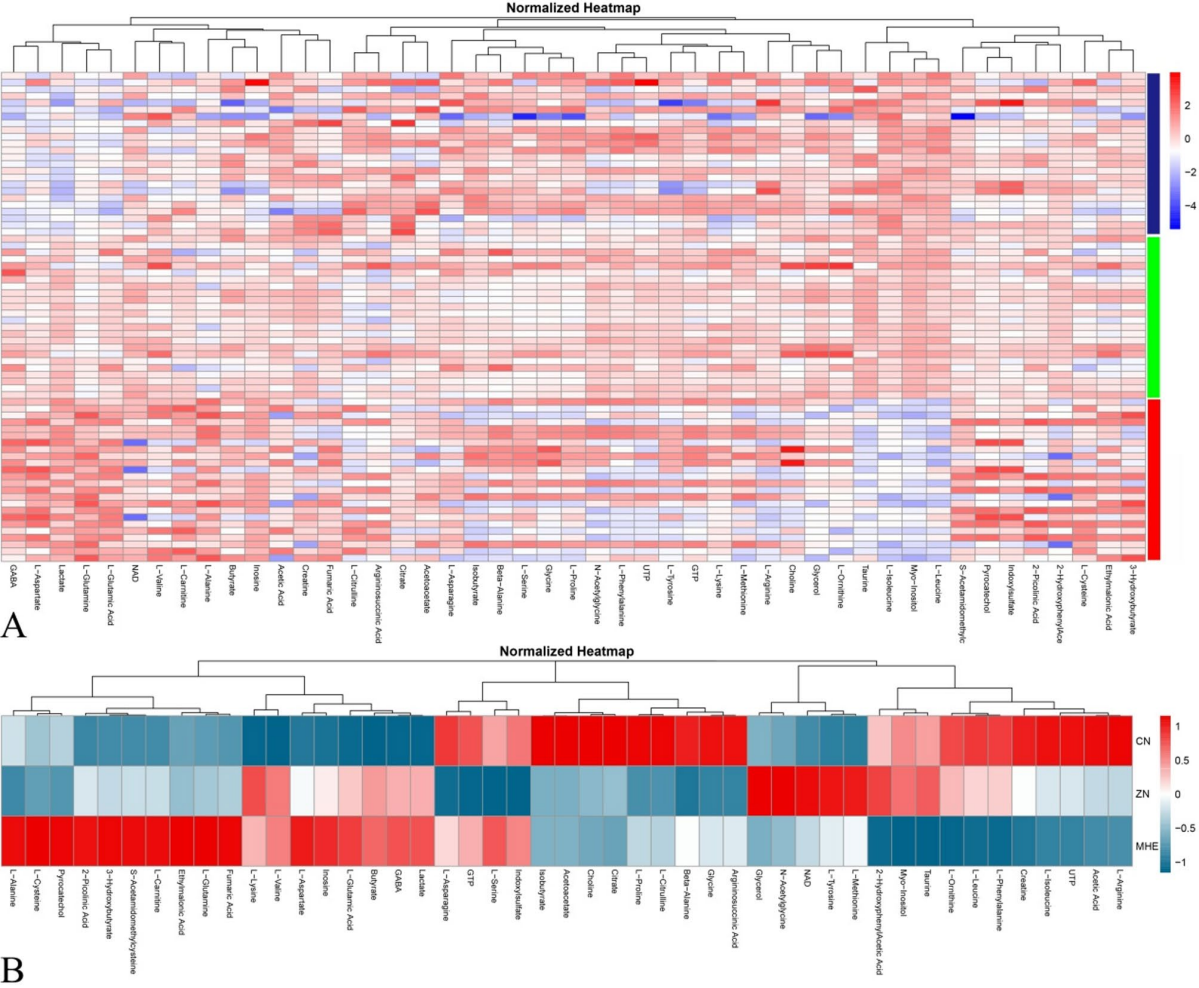
Heatmap of normalized metabolite levels. The metabolite levels is showed (**A**) across samples from minimal hepatic encephalopathy (MHE, rad bar), zinc-supplemented (ZN, green bar), and control (CN, blue bar) rats and (**B**) across groups (mean values of each group is used). The heatmap displays 47 metabolites identified through ^1^H-NMR spectroscopy after data preprocessing. Metabolites are arranged along the X-axis, while individual samples are represented on the Y-axis. The color scale ranges from blue (low concentration) to red (high concentration), indicating relative metabolite levels.

After preprocessing the ^1^H-NMR spectroscopy data from MHE, ZN, and CN rats, a total of 47 metabolites were identified for further analysis. These metabolites are visualized in a heatmap with hierarchical clustering (Fig. 2).

### Key metabolites selection

**Fig. 3:**
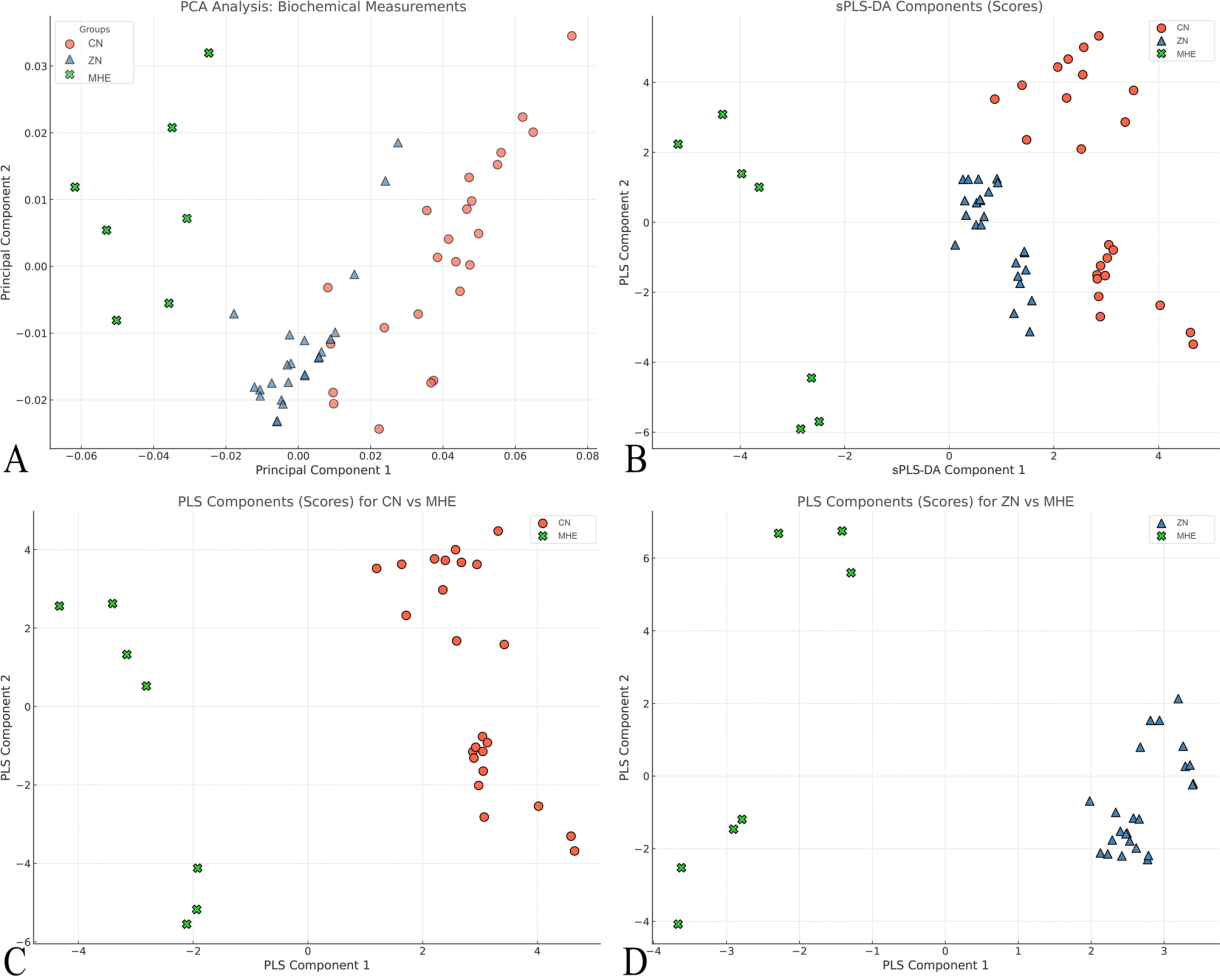
Multivariate analysis of metabolomic data from control (CN), minimal hepatic encephalopathy (MHE), and zinc-supplemented (ZN) rat groups. (**A**) Principal component analysis (PCA) plot showing the distribution of samples based on biochemical measurements. (**B**) Sparse partial least squares discriminant analysis (sPLS-DA) plot demonstrating clear separation among all three groups. (**C**) sPLS-DA plot comparing CN and MHE groups (R^2^ = 0.98, Q^2^ = 0.95). (**D**) sPLS-DA plot comparing ZN and MHE groups (R^2^ = 0.99, Q^2^ = 0.95). The plots reveal distinct metabolic profiles for each experimental group, with strong separation between MHE and both CN and ZN groups, indicating significant metabolic alterations in MHE and the impact of zinc supplementation. The high R^2^ and Q^2^ values for the sPLS-DA models suggest robust and predictive differentiation between the groups based on their metabolite profiles.

The metabolites among the three groups were well separated by both PCA and sPLS-DA analysis (Fig. 3). The metabolites between MHE and CN rats (R^2^ = 0.98, Q^2^ = 0.95) and between MHE and ZN rats (R^2^ = 0.99, Q^2^ = 0.95) were well separated by sPLS-DA analysis (Fig. 3). The fold change P-values and VIP scores are shown in Table 2.

**Table 2:** Key metabolites identified in CN ZN and MHE rats.

Metabolite	Fold change*	Fold change#	*p*-value*	*p*-value#	VIP*	VIP#
GABA	1.11	1.15	0.005	< 0.001	2.0	0.2
Myo-inositol	1.02	0.79	0.003	< 0.001	2.3	2.9
Lactate	1.28	1.48	0.005	< 0.001	2.2	1.6
Aspartate	1.16	1.42	0.002	< 0.001	1.3	1.0
Glutamine	1.07	1.57	0.003	< 0.001	2.4	2.7
Alanine	0.98	1.17	0.002	< 0.001	1.6	1.9
Taurine	0.96	0.65	0.002	< 0.001	1.8	2.5
Leucine	0.98	0.83	0.002	< 0.001	2.2	2.5
Glutamate	1.22	1.37	0.002	< 0.001	1.9	0.9
Isoleucine	0.93	0.82	0.002	< 0.001	2.4	2.3
Glycerol	1.08	0.98	0.002	< 0.001	0.0	1.1

*MHE vs. CN; # MHE vs. ZN; (fold change > 1 = increase in MHE; < 1 = decrease in MHE)

**Fig. 4:**
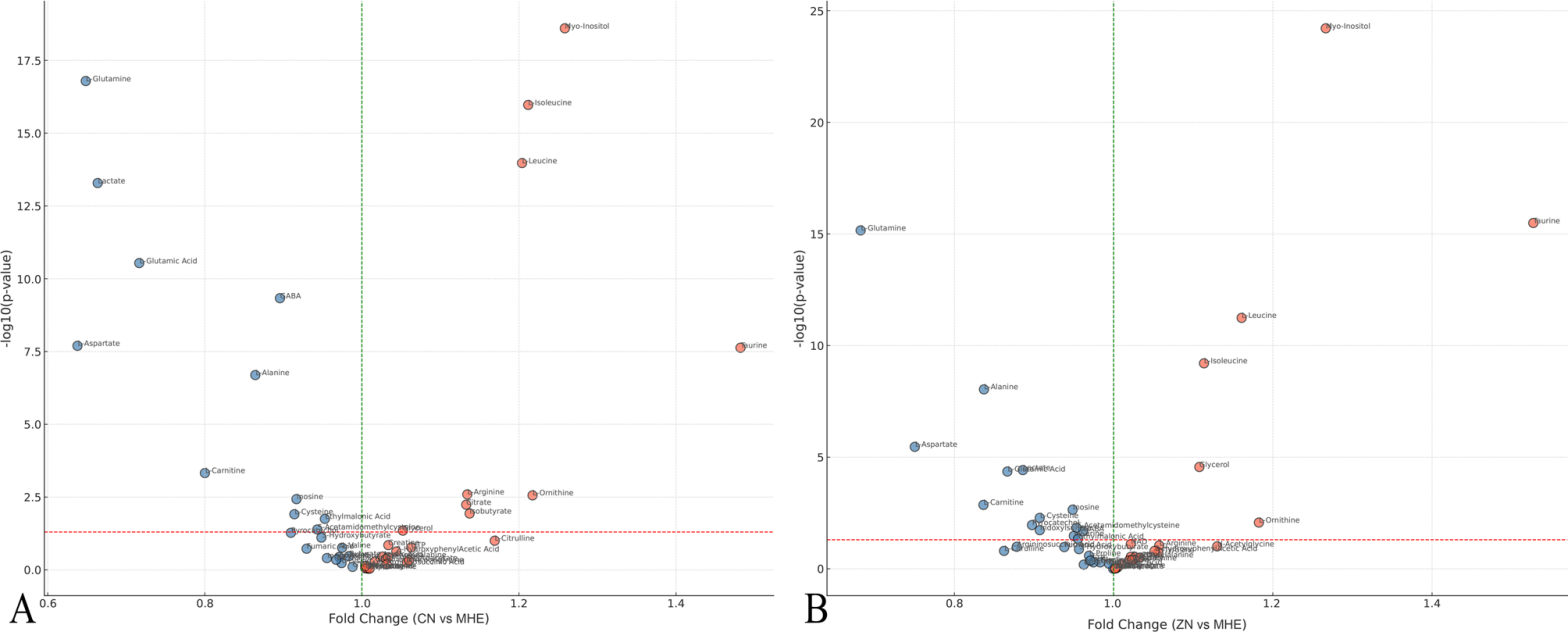
Volcano plots illustrating key metabolite changes in (**A**) MHE rats compared to CN rats, and (**B**) ZN rats compared to MHE rats. The x-axis represents the fold change in metabolite concentrations, while the y-axis shows the -log10(p-value). Each point represents a metabolite, with those above the horizontal dotted line considered statistically significant (*p* < 0.05). Red points indicate metabolites with increased concentrations, while blue points show decreased concentrations.

Differential analysis identified 10 key metabolites with significant changes, including increased concentrations of GABA, Myo-inositol, Lactate, Aspartate, Glutamine, Alanine, and Glutamate, and decreased concentrations of Taurine, Leucine, and Isoleucine between MHE and CN rats (Fig. 4).

Differential analysis identified 9 key metabolites with significant changes, with increased concentrations of Myo-inositol, Taurine, Leucine, Isoleucine, and Glycerol, and decreased concentrations of Lactate, Aspartate, Glutamine, and Alanine between MHE and ZN rats (Fig. 4). Notably, zinc supplementation markedly reduced the elevated lactate and alanine levels observed in MHE rats, restoring them toward control levels. This indicates that zinc mitigates the glycolytic dysregulation and impaired energy metabolism characteristic of MHE.

### Enrichment and pathway analysis

**Fig. 5:**
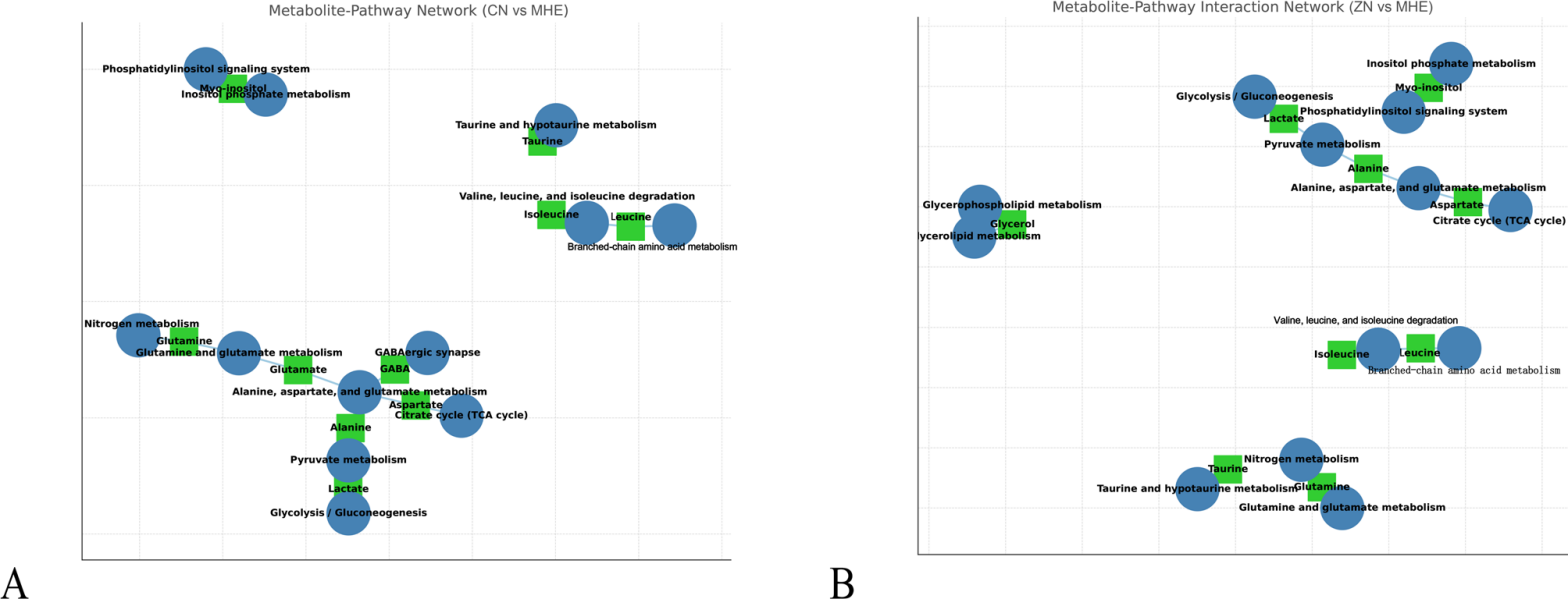
Metabolite pathway network (**A**) Pathway analysis of the ten key metabolites identified in MHE vs. CN rats revealed significant involvement of several metabolic pathways. These pathways included GABAergic synapse, inositol phosphate metabolism, glycolysis/gluconeogenesis, alanine, aspartate, and glutamate metabolism, glutamine and glutamate metabolism, taurine and hypotaurine metabolism, valine, leucine, and isoleucine degradation, and glycerolipid metabolism. (**B**) Zinc supplementation modulates the pathways of inositol phosphate metabolism, glycolysis/gluconeogenesis, alanine, aspartate, and glutamate metabolism, glutamine and glutamate metabolism, taurine and hypotaurine metabolism, valine, leucine, and isoleucine degradation, and glycerolipid metabolism in MHE vs. ZN Rats. The blue circles represent the metabolites, while the green squares indicate the pathways. The size of the circles and squares corresponds to the significance of the metabolite or pathway in the analysis.

Pathway analysis of the ten key metabolites from MHE vs. CN rats revealed involvement of GABAergic synapse, Inositol phosphate metabolism, Glycolysis/Gluconeogenesis, Alanine, aspartate, and glutamate metabolism, Glutamine and glutamate metabolism, Taurine and hypotaurine metabolism, Valine, leucine, and isoleucine in MHE metabolism. Pathways including Inositol phosphate metabolism, Glycolysis/Gluconeogenesis, Alanine, aspartate, and glutamate metabolism, Glutamine and glutamate metabolism, Taurine and hypotaurine metabolism, Valine, leucine, and isoleucine degradation, Glycerolipid metabolism were significantly enriched (*p* < 0.05) following zinc supplementation (Fig. 5).

## Discussion

This study employed ^1^H-NMR metabolomics to investigate key metabolite differences among MHE rats, zinc sulfate-treated MHE rats (ZN), and control (CN) rats. The findings highlight cognitive impairment and abnormalities in glycolysis, glutamine, and branched chain amino acids (BCAAs) metabolism in MHE rats. Zinc sulfate supplementation was observed to mitigate these impairments. Several metabolites closely associated with the TCA cycle, including lactate, alanine, and aspartate, showed significant alterations among groups. MHE rats exhibited elevated lactate and alanine levels and reduced aspartate levels, indicating impaired oxidative metabolism and increased reliance on anaerobic glycolysis. Zinc supplementation reversed these trends, restoring metabolite levels toward those of control rats.

Zinc deficiency is prevalent in MHE patients and is associated with elevated serum ammonia levels^13^. Zinc crosses the blood-brain barrier primarily through ZIP (Zrt-/Irt-like) transporters and DMT1 (divalent metal transporter 1)-mediated uptake, facilitated by metallothionein. These pathways enable regulated zinc entry into neuronal and glial cells. Zinc supplementation has been shown to decrease ammonia levels in both animal models and human studies^15^. Meta-analyzes have demonstrated significant cognitive improvements with zinc supplementation in cirrhotic patients with MHE^16^. Our behavioral assessments demonstrated significant cognitive impairment in MHE rats. The findings are consistent with previous studies showing spatial learning and memory deficits in MHE animal models^7,10^. Consistent with these clinical and animal studies, our results indicate that zinc sulfate restores cognitive function in MHE rats, as evidenced by improvements in escape latencies^7^. The cognitive benefits observed in our study parallel the metabolic normalization detected through NMR spectroscopy, suggesting that zinc’s therapeutic effects are mediated through restoration of disrupted brain metabolism.

Zinc is known to modulate nitrogen metabolism^17^ and influence ammonia levels. While some studies have not shown significant reductions in fasting plasma ammonia with zinc treatment in cirrhotic patients^15^. Our findings suggest that zinc supplementation affects pathways involving lactate and alanine, which are products of glycolysis^18^. The elevated lactate and alanine levels observed in our MHE rats indicate disrupted energy metabolism and enhanced glycolysis. These findings are consistent with Bosoi et al. (2014), who reported increased brain lactate as central to brain edema development in rats with chronic liver disease^18^. The accumulation of lactate reflects a shift toward anaerobic metabolism and mitochondrial dysfunction in the MHE brain^19^. Elevated alanine, a product of glycolysis and transamination reactions, further supports the presence of altered energy metabolism in MHE^18^. Our pathway analysis confirmed significant enrichment of glycolysis/gluconeogenesis pathways, reinforcing the central role of energy metabolism disruption in MHE pathophysiology. This normalization of lactate, alanine, and aspartate following zinc supplementation indicates a partial restoration of the TCA cycle’s metabolic balance, suggesting that zinc supports improved mitochondrial oxidative metabolism and energy production in the MHE brain.

Combined zinc and BCAA supplementation has been effective in reducing blood ammonia levels more than BCAAs alone in HE^20,21^. We observed significantly decreased levels of leucine and isoleucine in MHE rat striatum. This finding is consistent with the Fischer ratio imbalance commonly reported in HE, where BCAAs are decreased relative to aromatic amino acids. BCAAs serve as nitrogen carriers and energy substrates in the brain, and their depletion may impair protein synthesis and neurotransmitter metabolism^20,21^. The decreased BCAA levels in our study align with clinical observations and support the rationale for BCAA supplementation in HE management.

We observed significantly elevated glutamate and glutamine levels in the striatum of MHE rats, consistent with the established pathophysiology of HE^22,23^. Zhang et al. (2010) demonstrated elevated glutamine/glutamate levels in the anterior cingulate cortex of cirrhotic patients using magnetic resonance spectroscopy^22^. The accumulation of these metabolites reflects impaired ammonia detoxification, as elevated blood ammonia drives increased glutamine synthesis in astrocytes through the glutamine synthetase pathway^24,25^. Glutamate and glutamine levels were significantly elevated in MHE rats but normalized following zinc supplementation. Zinc’s normalization of glutamate and glutamine supports its role in mitigating ammonia-induced neurotoxicity and astrocytic swelling. Zinc sulfate supplementation was associated with decreased striatum glutamate and glutamine levels in MHE rats, indicating a regulatory role in these processes. In HE, zinc supplementation enhances urea cycle enzyme activity, improves hepatic ammonia clearance, and modulates neurotransmitter balance by influencing glutamate-glutamine cycling^15,20^.

This study has several limitations. First, the absence of a sham-operated plus zinc supplementation group restricts the full interpretation of zinc’s direct metabolic effects in non-MHE animals. Second, metabolomics analysis focused solely on the striatum while MHE affects multiple brain regions, so other areas like the cortex and hippocampus should be examined. Third, metabolites were measured at a single time point after 56 days of zinc supplementation, necessitating longitudinal metabolomics studies to understand temporal dynamics. Fourth, the rat model of MHE induced by partial portal vein ligation may not fully represent human MHE pathophysiology. Further validation in other animal models and clinical studies to enhance translational relevance is needed. Furthermore, water consumption was lower in zinc-supplemented rats, possibly due to reduced palatability or altered thirst regulation, which may have affected zinc intake and hydration. Although doses were adjusted weekly based on consumption, this could introduce variability in zinc exposure. Future studies should consider alternative methods like oral gavage for consistent dosing. In addition, only blood ammonia was measured in this study. The absence of urea, uric acid, and creatinine measurements limits the assessment of broader nitrogen and renal metabolism. Future studies should incorporate these parameters for a more comprehensive biochemical profile.

## Conclusions

In conclusion, zinc sulfate effectively improves cognitive impairments in MHE rats by restoring key metabolites related to alanine, glutamate, and branched-chain amino acid metabolism, thereby supporting improved energy metabolism and nitrogen balance in the brain.

## Data Availability

The datasets used and/or analyzed during the current study are available from the corresponding author on reasonable request.
